# Interactions of Isoquinoline Alkaloids with Transition Metals Iron and Copper

**DOI:** 10.3390/molecules27196429

**Published:** 2022-09-29

**Authors:** Mst Shamima Parvin, Jakub Chlebek, Anna Hošťálková, Maria Carmen Catapano, Zuzana Lomozová, Kateřina Macáková, Přemysl Mladěnka

**Affiliations:** 1 Department of Pharmacognosy and Pharmaceutical Botany, Faculty of Pharmacy in Hradec Králové, Charles University, Heyrovského 1203, 500 05 Hradec Králové, Czech Republic; 2 SwissBioquant, Kagenstrasse 18, 4153 Reinach, Switzerland; 3 Department of Pharmacology and Toxicology, Faculty of Pharmacy in Hradec Králové, Charles University, Heyrovského 1203, 500 05 Hradec Králové, Czech Republic

**Keywords:** alkaloid, iron, copper, chelation, reduction

## Abstract

Data on alkaloid interactions with the physiologically important transition metals, iron and copper, are mostly lacking in the literature. However, these interactions can have important consequences in the treatment of both Alzheimer’s disease and cancer. As isoquinoline alkaloids include galanthamine, an approved drug for Alzheimer’s disease, as well as some potentially useful compounds with cytostatic potential, 28 members from this category of alkaloids were selected for a complex screening of interactions with iron and copper at four pathophysiologically relevant pH and in non-buffered conditions (dimethyl sulfoxide) by spectrophotometric methods in vitro. With the exception of the salts, all the alkaloids were able to chelate ferrous and ferric ions in non-buffered conditions, but only five of them (galanthine, glaucine, corydine, corydaline and tetrahydropalmatine) evoked some significant chelation at pH 7.5 and only the first two were also active at pH 6.8. By contrast, none of the tested alkaloids chelated cuprous or cupric ions. All the alkaloids, with the exception of the protopines, significantly reduced the ferric and cupric ions, with stronger effects on the latter. These effects were mostly dependent on the number of free aromatic hydroxyls, but not other hydroxyl groups. The most potent reductant was boldine. As most of the alkaloids chelated and reduced the ferric ions, additional experimental studies are needed to elucidate the biological relevance of these results, as chelation is expected to block reactive oxygen species formation, while reduction could have the opposite effect.

## 1. Introduction

Although iron and copper have a number of physiological functions in the human body, their dyshomeostasis has been observed in several diseases, including cardiovascular diseases, cancer and some neurodegenerative disorders, including Alzheimer’s disease [1,2,3]. The treatment of the latter disease is still largely insufficient; notwithstanding the large amount of pharmacological and technological testing [4,5] the number of FDA-approved drugs is limited to four small molecules and one partly controversial monoclonal antibody [6]. The isoquinoline alkaloid, galanthamine, is one of these approved drugs. Its major mechanism of action is the inhibition of acetylcholinesterase. Several of its congeners were also experimentally proven to block this enzyme [7,8,9]. Hence, it is of interest to test this alkaloid together with its structurally related isoquinoline congeners for their interactions with these two biogenic metals. These interactions can have either positive or negative consequences. In the case of Alzheimer’s disease, the chelation or redistribution of transition metals appears to be positive, while reduction can enable the formation of hydroxyl radicals via the Fenton reaction; it is therefore potentially associated with additional damage. In cancer, the situation is different and many of these alkaloids were shown to possess cytostatic effects. As tumors grow rapidly, they require a higher influx of both iron and copper and, hence, both the chelation and the reduction of these metals lead to cytostatic effects [10,11,12].

Notwithstanding that many papers reported the antioxidant effect of alkaloids [13], the data on metal interactions are much more limited [14,15,16,17,18,19,20,21]. This might be explained by the fact that most alkaloids based on their structure were formerly supposed to be free of chelation activity. Previous studies, however, observed repeatedly unexpected iron-chelating effects in some alkaloids with isolated hydroxyl groups. Examples of this were found in alkaloids with different structural forms, e.g., phenanthrenes [18], morphinanes [22] and, in particular, several isoquinoline alkaloids [17,18,22,23], as well as their bisbenzylisoquinoline congeners [19,22,24]. In some cases, these alkaloids were even similarly potent to the known chelator, ethylenediaminetetraacetic acid (EDTA), employed in these studies as the positive control [18,19]. On the other hand, it should be mentioned that the same alkaloids also reduced the levels of ferric ions. Data on copper’s interactions with alkaloids are very rare in the literature [15,16,19,21]. One scarce example is the tetrahydroisoquinoline alkaloid salsolinol, which causes profound damage to DNA and cells when mixed with copper. The potentiation of the copper-based Fenton chemistry was demonstrated, but the chelation or reduction of the copper was not measured [21]. In general, most of the relevant studies included the interaction of alkaloids with copper and iron as a part of research related to antioxidant effects. A profound analysis and detailed comparison of the chelation and reduction activity of alkaloids is lacking.

For this reason, the aim of the study was to assess the interaction of 28 isoquinoline alkaloids (Supplementary Data Figure S1) with iron and copper in detail at different pathophysiologically relevant pH, from an acidic pH of 4.5 to a neutral pH, in non-buffered conditions and compare their effects with clinically used standard chelators. The novelty of this paper is a comparison of a large row of structurally related alkaloids in relation to two major biological redox-active transition metals together with testing of the impact of pH, which has not been previously reported. This paper confirmed previous isolated findings in non-buffered conditions and emphasizes the need of testing under different pH conditions, as the selection of pH markedly affected the results, particularly in relation to the non-buffered conditions.

## 2. Results

### 2.1. Iron Chelation in Non-Buffered Conditions: All Tested Alkaloids Chelated iron

**Figure 1:**
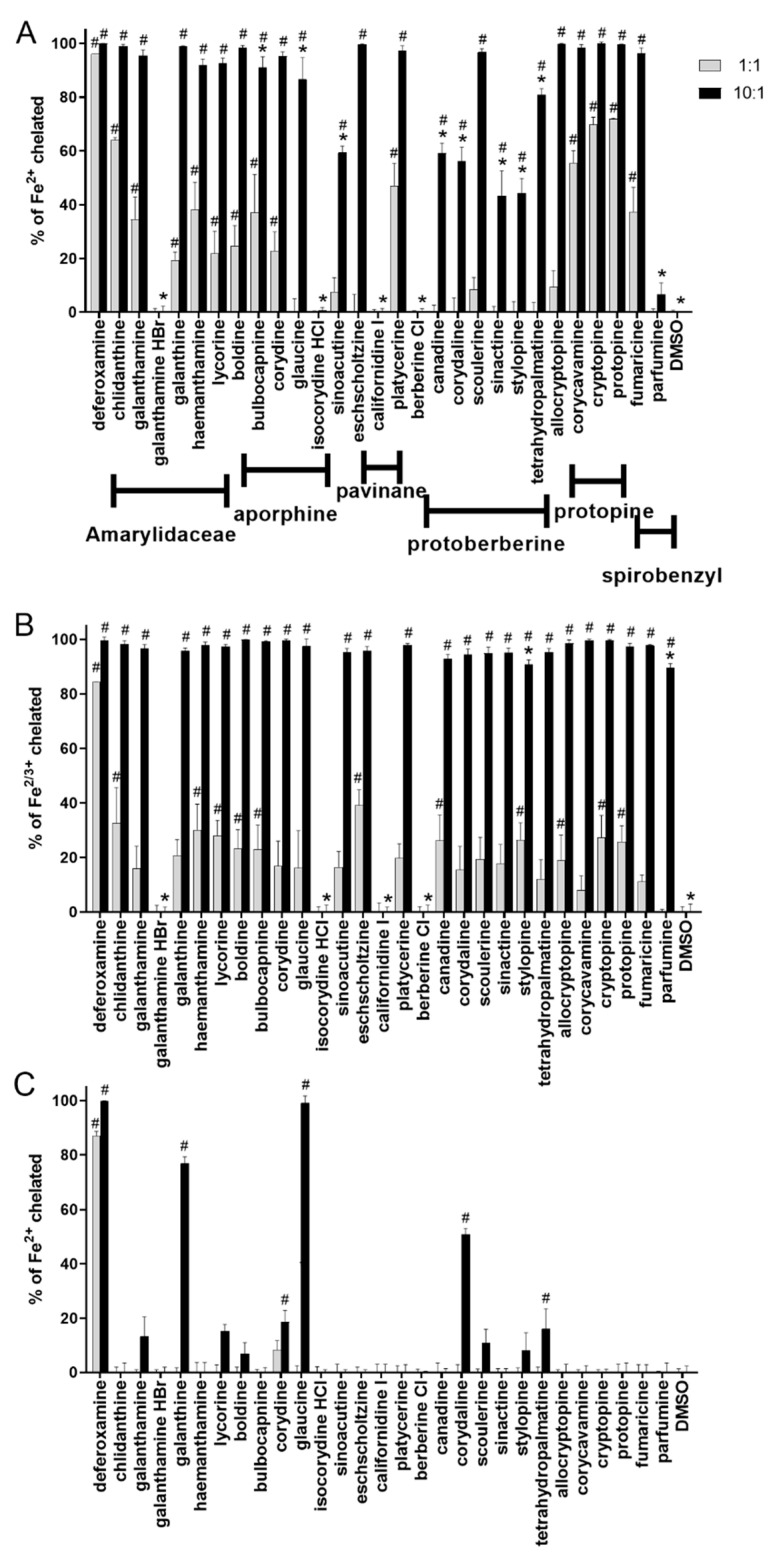
Iron-chelation efficiency of alkaloids in ratios of 1:1 and 10:1 (alkaloid:iron) in comparison with the clinically used iron chelator deferoxamine. (**A**) Ferrous chelation in non-buffered conditions; (**B**) Total iron (ferric + ferrous) chelation in non-buffered conditions; (**C**): Ferrous chelation at pH 7.5: Chelation efficiency of all substances was significantly different from deferoxamine/not shown in the graph/except glaucine at a ratio of 10:1. At a 1:1 ratio, all substances were significantly less potent than deferoxamine under all conditions at *p* < 0.001. * vs. deferoxamine at *p* < 0.001, # vs. solvent (dimethylsulfoxide, DMSO) at *p* < 0.001. Sinoacutine is a morphinane alkaloid.

In the first step, all the alkaloids were screened for their ability to chelate iron ions in a non-buffered environment using the ferrozine spectrophotometric method, which is based on competition between a tested alkaloid and ferrozine for free (non-chelated) iron [25]. All the alkaloids in the form of free bases were able to chelate both ferrous and ferric ions, although to a lesser extent than the clinically used standard iron chelator deferoxamine (Figure 1). However, in a compound-to-iron-ion concentration ratio of 10:1, many of the alkaloids chelated practically all the added metal ions, as did deferoxamine. The effect was more pronounced in the case of total iron chelation (Figure 1B), where all the tested alkaloids in the form of free bases achieved complete chelation with one exception, parfumine. This suggested a higher affinity for ferric ions, but it should also be mentioned that this effect was observed solely at higher ratios. By contrast, at the equimolar (1:1) ratio, four alkaloids (chlidanthine, corycavamine, cryptopine and protopine) chelated more than 50% of the ferrous ions, but apparently fewer ferric ions. Importantly, all the chelating alkaloids formed stable complexes, whose stability was mostly in the range of 90–100% (Supplementary Figure S2).

### 2.2. Ferrous Chelation under Different pH Conditions: Only Galantine and Glaucine Were Active at Slightly Acidic pH 6.8

**Figure 2:**
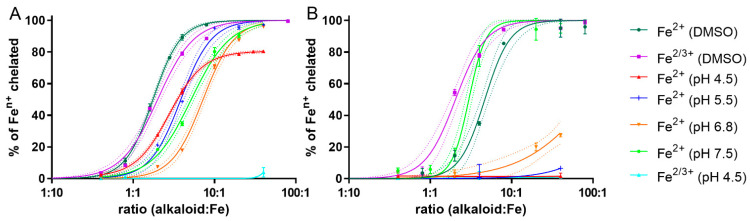
Iron-chelation activity of galanthine (**A**) and glaucine (**B**) under different conditions.

In the next step, chelation was tested in buffered conditions at four pathophysiologically relevant pHs, ranging from 4.5 to 7.5. In general and in contrast to the non-buffered conditions, only 5 alkaloids, galanthine, glaucine, corydine, corydaline and tetrahydropalmatine, evoked significant chelation at pH 7.5 at a rate of 10 times that of the ferrous ions. However, only two, galanthine and glaucine, were also active at pH 6.8. Galanthine (Figure 2A) was the sole alkaloid that chelated the ferrous ions significantly under all the pH conditions. Another alkaloid, glaucine, was even more potent, at pH 7.5, than the galanthine, but its effect markedly dropped with the pH and was absent at the two lowest pH (Figure 2B).

### 2.3. Confirmation of the Ferric Chelation Effect of Glaucine in Non-Competitive Conditions

**Figure 3:**
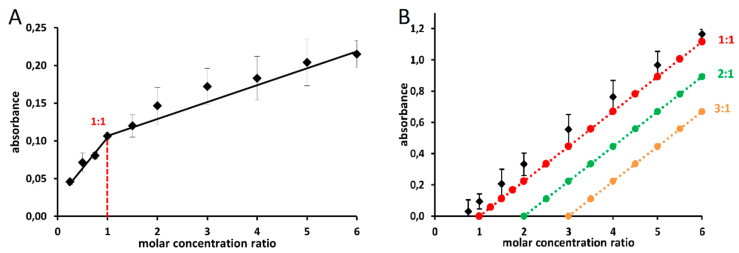
Stoichiometric determination of the complex glaucine:Fe^3+^ at pH 7.5 by the Job’s plot (**A**) and the complementary method (**B**). The suggested stoichiometry of the complex was 1:1. Data are from two independent experiments and are depicted as means and standard deviations.

In order to verify whether the most active alkaloid glaucine could also chelate the ferric ions, a non-competitive approach was used. Firstly, the spectra of glaucine and its mixture with ferric ions were compared. Under all the tested pH, as well as in the non-buffered conditions, the addition of ferric ions clearly shifted one peak in the spectra of the glaucine, suggesting a complex formation (Supplementary Data Table S1 and Figure S3). The stoichiometry of the complex was also tested at pH 7.5 and the complex 1:1 was observed in both the employed methods (Figure 3).

### 2.4. Copper Chelation: None of the Tested Alkaloids Significantly Chelated Cupric or Cuprous Ions

After determining the iron-chelation activity, the copper chelation was tested by two competitive methods, with hematoxylin and bathocuproinedisulfonic acid disodium salt (BCS). However, no cupric or cuprous chelation was observed in either of these assays, in contrast to the clinically used copper chelator, trientine (data not shown).

### 2.5. Metal Reduction: Most Alkaloids Reduced Ferric and, in Particular, Cupric Ions

The ferrozine and BCS methods were also used for the determination of the metal reduction activity of the alkaloids. With the exception of the protopine alkaloids, all the other tested compounds were shown to be active reductants, although their potency was very different. Their effect was especially strong in the case of the copper ions (Supplementary Table S2).

**Figure 4:**
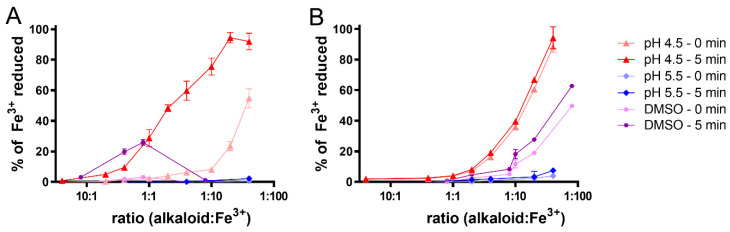
Iron-reduction activity determined immediately after measurement (time 0 min) and after 5 min. (**A**) scoulerine; (**B**) glaucine. No reduction was observed at pH 6.8 or 7.5.

The iron was reduced under both non-buffered conditions, as well as at pH 4.5. With the exception of a very mild ferric reduction by glaucine at pH 5.5, none of the tested alkaloids were able to reduce the ferric ions at pH 5.5 or under higher-pH conditions. There were also apparent differences between the ferric reduction at pH 4.5 and in the unbuffered conditions. In line with the chelation data, the reduction at pH 4.5 was more pronounced than in the non-buffered conditions. In the formed case, the reduction activity increased with the increasing alkaloid:iron ratio (Figure 4B). The most active substances, isocorydine, glaucine, sinoacutine and scoulerine, showed almost complete iron reduction at the highest tested ratio.

In the unbuffered conditions, there were six alkaloids with bell-shaped reduction curves. Four aporphine alkaloids (boldine, bulcocapnine, corydine and isocorydine), sinoacutine and scoulerine achieved the highest reduction, at ratios of approximately 1:1 to 2:1, while there was a decrease in the reduction activity at higher ratios (bell-shaped curves, Figure 4A).

In order to determine the structure–activity relationships, the linear regression lines based on the relationship between the percentage of reduced iron and the concentration ratio of the alkaloids to ferric ions were constructed in ratios associated with progressive and significant reduction. Their 95% confidence intervals were then compared. An example comparison is shown in Supplementary Data Figure S4A and a schematic comparison of the activity of the most potent alkaloids is presented in Supplementary Data Figure S5. Five of the alkaloids were able to reduce the ferric ions at pH 4.5 at ratios below 1:1, alkaloid:iron. The most potent compound was boldine, followed by bulbocapnine, isocorydine, scoulerin and corydine. Concerning the structure–activity relationship, a reasonable comparison could be performed only within the aporphines and protoberberines, as the alkaloids from the other subgroups were only mildly active ferric reductants. Among the aporphines, the reduction effect was the same from the statistical point of view for all the alkaloids with a bell-shaped effect (boldine, bulcocapnine, corydine and isocorydine) under non-buffered conditions while glaucine started to reduce the ferric ions at much higher ratios. At pH 4.5, the differences were more pronounced. However, from the statistical point of view, there were no differences between boldine, bulbocapnine and isocorydine. The corydine was less potent, while glaucine had the weakest effect. In terms of the protoberberines, scoulerine was clearly the most potent, followed by berberine. All the other tested representatives (canadine, corydaline, sinactine, stylopine and tetrahydropalmatine) had the same, only mild reducing, effect.

**Figure 5:**
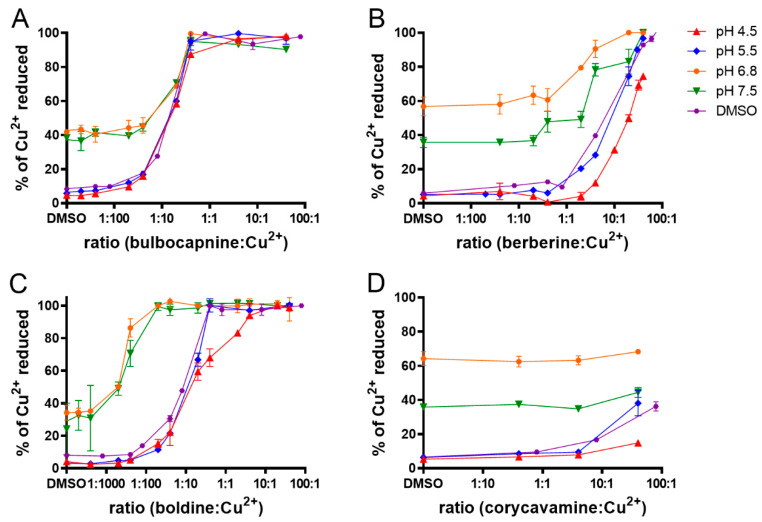
Examples of the reduction curves showing the relationship between the molar concentration ratio (alkaloid:Cu^2+^) and the copper reduction activity at 5 min. (**A**): bulbocapnine; (**B**): berberine; (**C**)**:** boldine; (**D**): corycavamine. The data represent means ± SD.

As mentioned above, the alkaloids’ cupric-reducing effects were stronger than those on the ferric ions. They were observed under all the tested pH conditions, as well as in non-buffered conditions. The aporphine alkaloids were found to be very significant copper reducers, achieving complete reduction at a ratio of 1:1 (alkaloid:copper), with the exception of glaucine under some conditions. The bulbocapnine and boldine were even more potent and achieved complete reduction at ratios of 1:10 and 1:50, alkaloid:Cu^2+^, respectively (Figure 5A–C). Among the other structural types, the Amaryllidaceae alkaloid, chlidanthine, morphinane alkaloid, sinoacutine, pavinane alkaloid, platycerine, protoberberine alkaloid, scoulerine and spirobenzylisoquinoline alkaloid, parfumine showed very significant but not complete activity at a ratio of 1:1. The other alkaloids exhibited no or only weak cupric-reduction activity at this ratio; however, their copper-reduction effect was significant at the highest tested ratios. A schematic comparison of the activities of the most potent alkaloids analyzed as reduction lines (Supplementary Data Figure S4B) is shown in Supplementary Data Figure S6. In general, the most potent reducing alkaloid was boldine, at pH 6.8 and 7.5, followed by platycerine. At pH 4.5 and 5.5, platycerine achieved the highest potency, followed by boldine, scoulerine and bulbocapnine.

**Figure 6:**
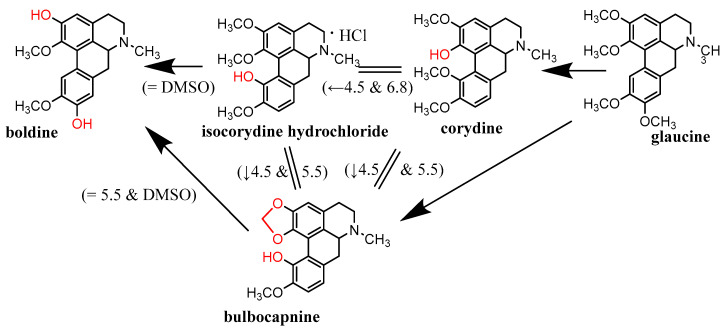
Graphical depiction of cupric-reduction potencies of tested aporphine alkaloids. Arrows point to stronger reduction compounds. Exceptions to the general scheme are shown in parentheses, including the conditions.

**Figure 7:**
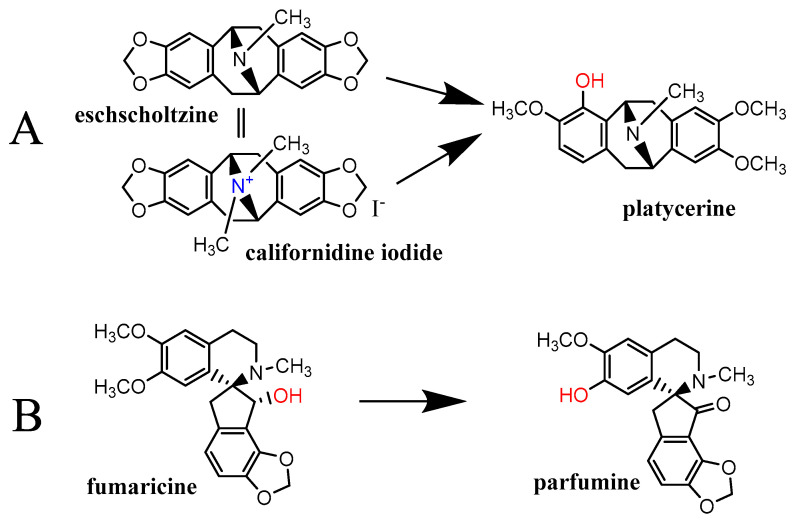
Graphical depiction of cupric-reduction potencies of tested pavinane alkaloids (**A**) and spirobenzylisoquinolines (**B**). Arrows point to stronger reduction compounds.

**Figure 8:**
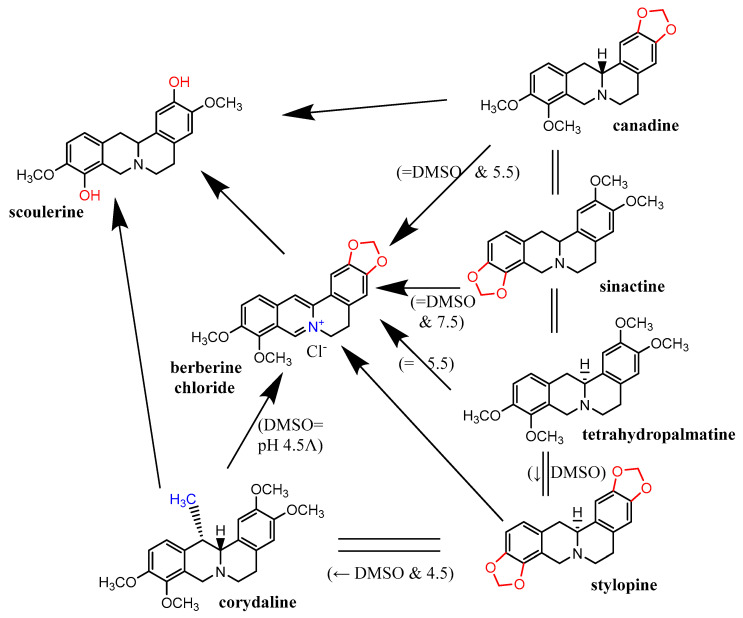
Graphical depiction of cupric-reduction potencies of tested protoberberine alkaloids. Arrows point to stronger reducing compounds. Exceptions to the general scheme are shown in parentheses, including the conditions. Ʌ—corydaline was a more potent reduction agent at lower ratios, while berberine was more potent at higher ratios.

**Figure 9:**
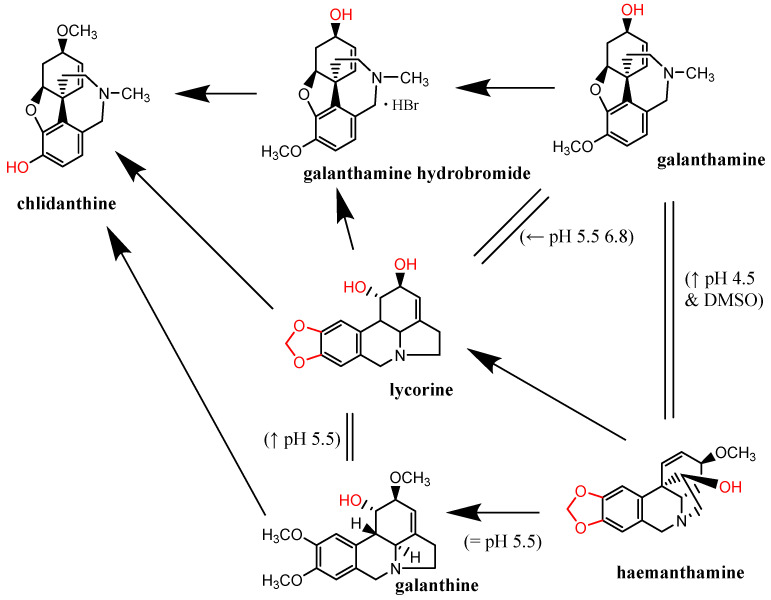
Graphical depiction of cupric-reduction potencies of alkaloids isolated from Amaryllidaceae family. Arrows point to stronger reduction compounds. Exceptions to the general scheme are shown in parentheses encompassing conditions.

As the cupric-reduction activities were more expressed than those for the ferric ions, a detailed activity-structure relationship was created. Graphical comparisons of the reduction activities in different subclasses are shown in Figure 6, Figure 7, Figure 8 and Figure 9. In the case of the aporphines, the reduction followed the number of free hydroxyl groups in general (Figure 6). The most potent was boldine, with two free hydroxyl groups, followed by isocorydine and corydine, each with one free hydroxyl group, and glaucine, without a free hydroxyl group. Bulbocapnine, which possessed a methylendioxy bridge in addition to one free hydroxyl group, was, in general, similarly potent to isocorydine and corydine, except at lower pH, where it was more active. A similar phenomenon was observed in the cases of the three tested pavinanes (Figure 7A). Under all the conditions, platycerine with one free hydroxyl group was more potent than other two compounds without free hydroxyls. Both the tested spirobenzylisoquinoline alkaloids possess one free hydroxyl group, but fumaricine has this hydroxyl on saturated cyclopentyl residue, while that in parfumine is aromatic, localized on the benzylisoquinoline ring. The comparison of these two compounds showed a clearly stronger reduction property of the phenolic (aromatic) hydroxyl (Figure 7B). In the case of the protoberberines (Figure 8), the strongest reduction agent was scoulerine, with two hydroxyl groups. Other compounds differing in the presence of a methylendioxy bridge or *ortho*-dimethoxy group had mostly similar potencies to berberine (this quaternary ammonium salt was only slightly more potent in most cases). The tested Amaryllidaceae alkaloids were not structurally homogenous, but the results confirmed and extended previous findings (Figure 9): the most potent compound from this group, chlidanthine, contained one free phenolic hydroxyl. Neither two free non-aromatic hydroxyl groups nor a partly unsaturated cyclohexen ring (lycodine) resulted in a more potent reduction agent. Again, the presence of a positively charged nitrogen slightly improved the activity (galanthamine vs. galanthamine hydrobromide), which was, however, low. Only negligible activity was observed in the case of hemanthamine, which had only one non-aromatic free hydroxyl.

## 3. Discussion

The currently available data on the interactions of alkaloids with iron and copper are largely insufficient. In addition, the understanding of this research is complicated given the limited number of studies on this topic, which, furthermore, merely touch on this topic rather than concentrating on it profoundly. There could be two major reasons for this: (1) many alkaloids were not supposed to possess metal chelation activity based on their structure (e.g., the absence of a clear chelation site, in contrast to the well described chelation groups in flavonoids [26]); and (2) the difficulties in publishing studies with negative or partly positive data. There are also some exceptions. For example, 3,8-dihydroxyquinoline isolated from *Scolopendra subspinipes* possesses adjacent aromatic nitrogen and a hydroxyl group [16], and this is a known chelation site [27]. However, most of the alkaloids that have been shown to chelate iron do not possess such a clear chelation site. In relation to the isoquinoline alkaloids, previous studies demonstrated that isocorydine, norisocorydine, coptisine, berberrubine, boldine, norboldine and (+)-*N*-methylisococlaurine chelated ferrous ions [17,18,20,22]. Isocorydine has only one free phenolic hydroxyl, while the nitrogen atom is blocked by a methyl group; norisocorydine has a free nitrogen atom, but there is no other chelation group in its vicinity. Both coptisine and berberrubine possess a methylendioxy group, while berberrubine also has one free phenolic hydroxyl. *N*-Methylisococlaurine has two free phenolic hydroxyls, but they are localized on different benzene rings. Boldine and norboldine are similar examples, with two free phenolic hydroxyls in an *ortho* position to the methoxy groups; they differ only in the absence or presence of a methyl group on the nitrogen atom. These data therefore represent an enigma for chemists, and clearly necessitate more profound physicochemical studies using more sophisticated instrumental approaches for the detection of the structures of the formed chelates. Nevertheless, based on previous results and the outcomes of this study, it is clear that alkaloids with hydroxy-methoxy groups in an *ortho* position, as well as those with a methylendioxy ring, or even those with an *ortho* dimethoxy group (such as galanthine and glaucine in our study) can chelate iron and their chelation properties can be fairly strong (e.g., Figure 2). As the *ortho* dimethoxygroup was not associated with chelation in coumarins [28], it is very probable that nitrogen atoms contribute to this process in alkaloids. The chelation pattern was markedly different when buffers were used. In particular, more acidic pH abolished the chelation effect of almost all the tested alkaloids. Hence, it is not surprising that alkaloids in the form of salts/hydrochloride, hydrobromide, chloride or iodide/were not active in this study in non-buffered conditions (Figure 1). As far as we know, these are highly novel data, as all the previous studies with alkaloids tested iron chelation only in solvents. There are also some discrepancies between the data from the literature and those in our study. Berberine and its structural congener were not able to chelate ferrous ions in one study [20]. There are some possible explanations for this phenomenon; the authors may have used salts, as we did with berberine, the solvent (not specified) may have not been suitable for iron-chelation testing, or the authors simply tested at low ratios; it appears from their graph that the highest ratio of alkaloid to ferrous salt was only 2:1. According to Figure 1 or even Figure 2 in our study, this ratio might not be sufficient to detect significant chelation. It should be also mentioned that one study claimed berberine to chelate iron but, based on the reported methodological approach and results, ferrous reduction took place instead of chelation [14].

In general, the survey of the currently available chelation and reduction data on alkaloids is also complicated by the fact that most of the relevant studies included this testing as a marginal part of their research; since they did not assign strong importance to it, the methodological data are not sufficient to calculate the concentration ratios between the alkaloid and the metal in several cases. In some of the studies, the authors included EDTA as a standard metal chelator, and therefore we can at least indirectly calculate the concentration ratio between alkaloid and the metal. Since EDTA chelates metals at a ratio of 1:1, the reported IC_50_ representing 50% of the chelated iron signifies an EDTA:metal ratio of 0.5:1. For example, the IC_50_ of isocorydine was 88 μM in one study, while that of EDTA was 20 μM [17]. Therefore, we can suppose that there was a need for an excess of about 4.5-fold of this alkaloid to chelate all the iron in the solution. This result is in agreement with our data on the isocorydine isomer, corydine, which chelated nearly all the ferrous ions in a 10-fold excess, while only about 20% of the iron was chelated in a ratio of 1:1 (Figure 1). Boldine was reported to be much less potent that EDTA, by approximately 11.5-fold [22], which is a lower potency than that observed in our study. By contrast, coptisine, an unsaturated congener of stylopine tested in our study, had a potency about 40% of that of EDTA at a 1:1 ratio, suggesting a copistine:ferrous-ion ratio of almost 2:1 [20]. Four alkaloids in our study chelated 50% or more ferrous ions in non-buffered conditions at a ratio of 1:1 (Figure 1A), suggesting the formation of an alkaloid:iron complex with a ratio of 2:1. It should be also emphasized that these complexes were stable in competition with the indicator ferrozine, which is also a potent ferrous chelator (Supplementary Data Figure S2). Hence, these data suggest that some of these alkaloids are relatively strong chelators.

Deciphering the biological relevance of the chelation data is complicated by the fact that most of the alkaloids included in this study also reduced both the copper and the iron ions. The only exceptions were the protopines, which were free of this effect in the range of the tested concentrations. It should be also mentioned that some of the included alkaloids were able to reduce the metal ions only at high ratios, particularly in relation to the ferric ions, and the extent was not particularly high. This mild reduction was observed with galanthamine, which is logical in light of possible negative consequences. This property could be associated with side effects during the treatment of Alzheimer’s disease, as the reduction in trace metals can potentiate the Fenton chemistry [10,11]. On the other hand, this effect is likely to be suitable for cancer treatment. This theory is largely based on the effect of other reducing compounds, but it is supported by few data in relation to alkaloids, e.g., salsolinol potentiated the Cu-based Fenton chemistry and increased cellular Cu-damage [21]. As some alkaloids are also antioxidants, they can react with formed hydroxyl radicals; therefore, depth studies are needed for the elucidation of the outcomes. This is highly important, as there are recent proofs in relation to flavonoids [29], which are both direct free-radical scavengers, metal chelators and metal reductants. Furthermore, the final outcome was difficult to predict without experimental confirmation. A similar phenomenon cannot be fully excluded in alkaloids. Studies with coptisine, berberrubine, palmatine and berberine showed that all these compounds reduced the hydroxyl-radical production within the iron-based Fenton reaction, albeit in different magnitudes [20]. There are many papers reporting iron and copper reduction by several alkaloids, some of which were even more or similarly potent to standard reductants (e.g., butyrated hydroxytoluene, butyrated hydroxyanisole, vitamin C, vitamin E, or its hydrophilic substitute Trolox) [17,18,19,22,24]. The final effect on metal-triggered cellular injury is, however, not known and must be proven experimentally in the future. By contrast, chelating alkaloids could, in line with the theory, block copper-based pro-oxidation, as was demonstrated by copper chelating 3,8-dihydroxyquinoline [16].

Our study has several strengths, as well as several limitations. It is the first study to have analyzed a relatively large group of alkaloids for their chelation and reduction effects on both copper and iron. Importantly, to the best of our knowledge the interactions were tested under different pH conditions and in non-buffered conditions for the first time in this study. We selected only one solvent (dimethylsulfoxide, DMSO) and this could also have had an impact as, for example, the reduction of ferric ions is markedly influenced by the selected solvent [28]. There is also a need to analyze the consequences of alkaloid–metal interactions in more biologically relevant conditions in the future, as the final anti- or prooxidative effect is difficult to assess. In addition, we only used spectrophotometric techniques, but as our results generally agree with the literature, we do not think the use of other methods will produce different data. However, more sophisticated methods are needed to determine the precise metal-binding sites and stoichiometry of the complexes.

## 4. Materials and Methods

### 4.1. Chemicals

Berberine Cl, isocorydine HCl, cupric sulfate pentahydrate (CuSO_4_ ∙ 5H_2_O), cuprous chloride (CuCl), DMSO, ferric chloride hexahydrate (FeCl_3_ ∙ 6H_2_O), ferrous sulfate heptahydrate (FeSO_4_ ∙ 7H_2_O), 3-(2-pyridyl)-5,6-diphenyl-1,2,4-triazine-4′,4′′-disulfonic acid sodium salt (ferrozine), hydroxylamine hydrochloride (hydroxylamine), hematoxylin, hydrochloric acid (HCl), BCS, sodium chloride (NaCl), sodium acetate, acetic acid, 4-(2-hydroxyethyl)-1-piperazineethanesulfonic acid (HEPES), HEPES sodium salt and trientine were purchased from Sigma-Aldrich (Czech Republic), while deferoxamine was from Novartis (Switzerland), boldine was from Carl Roth (Germany), glaucine was from Cayman Chemical (Ann Arbor, MI, USA) and galanthamine hydrobromide was from Changsha Organic Herb (Changsha, China). Chlidanthine and galanthamine were isolated from *Chlidanthus fragrans* herb [30], haemanthamine, lycorine and galanthine from *Zephyranthes robusta* (Herb. ex Sweet) [31], bulbocapnine, corydine, sinoacutine, canadine, corydaline, scoulerine, tetrahydropalmatine, allocryptopine and corycavamine from *Corydalis cava* (L.) [32], californidine iodide, eschscholtzine and protopine from *Eschscholia californica* [33], platycerine from *Argemona platyceras* [34] and sinactine, stylopine, cryptopine, fumaricine and parfumine from *Fumaria officinalis* L. [35].

### 4.2. Iron/Copper Chelation-and-Reduction Determination

All experiments were carried out on SynergyTM 2 Multi-Mode Microplate Reader (BioTec Instruments, Inc., Omaha, NE, USA) in 96-well microplates with the use of methodologies described previously [28,36]. Deferoxamine was used as a positive control for iron chelation, while trientine was used for copper chelation.

#### 4.2.1. Reagent and Stock-Solution Preparation

The stock solutions of ferric (FeCl_3_ ∙ 6H_2_O), ferrous (FeSO_4_ ∙ 7H_2_O) and cupric (CuSO_4_ ∙5H_2_O) ions were prepared in distilled water (Milli-Q RG, Merck Millipore, Burlington, MA, USA), as well as hydroxylamine, ferrozine and BCS solutions. The stock solution of cuprous (CuCl) ions was prepared by dissolution in an aqueous solution of 0.1 M HCl and 1 M NaCl. The solutions of tested alkaloids and hematoxylin were prepared in DMSO. All experiments were performed under non-buffered and buffered conditions (15 mM acetate buffers for pH 4.5 and 5.5, 15 mM HEPES buffers for pH 6.8 and 7.5).

#### 4.2.2. Ferrozine Assay

Briefly, various concentrations of tested alkaloids were mixed with ferrous or ferric ions. The color reaction was initiated by addition of ferrozine. In buffered conditions, the buffers were added prior to the tested samples. Ferrozine is the indicator of ferrous ions. Therefore, hydroxylamine was added in case of total iron chelation to reduce non-chelated ferric ions to ferrous ions. The absorption was measured immediately after the addition of ferrozine (0 min) and 5 min later. All measurements were performed at least in duplicates. Deferoxamine was used as a positive control.

The same methodology was used for determination of ferric ion reduction. Hydroxylamine was used as a positive control, causing 100% ferric-ion reduction.

#### 4.2.3. Hematoxylin Assay

The alkaloid DMSO solutions were mixed with various buffers and cupric-ion solution and after 2 min of shaking, hematoxylin or DMSO (blank sample) was added. The absorbance was measured after 3 min of shaking and later after additional 4 min at different wavelengths, depending on the tested pH, as reported previously [36].

#### 4.2.4. BCS Assay

This assay was identical to the ferrozine assay, except for the use of the BCS reagent specific to cuprous ions. Briefly, alkaloid solutions were mixed with cupric or cuprous solutions and incubated for 2 min with or without buffer. Hydroxylamine was added prior to cuprous ions to keep them in reduced form or after the mixing with cupric ions to reduce non-chelated cupric ions to cuprous ions. The amount of non-chelated copper was determined spectrophotometrically after the addition of the BCS reagent.

As in the case of the ferrozine method, this method is also suitable for determining the copper-reducing capabilities of test substances. Again, hydroxylamine was employed for complete reduction of cupric ions.

#### 4.2.5. Assessment of Iron Stoichiometry

The stoichiometry was assessed according to the previously published methodology [37]. The methanolic solutions of alkaloids were mixed with iron ions at different molar ratios, from 1:4 to 6:1, at all pH conditions. The absorbance was measured immediately after 1 min of shaking by Helios Gamma spectrophotometer equipped with VISIONlite software 2.2 (ThermoFisher Scientific Inc., Waltham, MA, USA). The blank sample consisted of buffer pH 7.5 and methanol in a ratio of 2:1. The measurements were performed in UV-transparent cuvettes (BrandTech Scientific Inc., Saint Neots, UK). Two methods were used to find and confirm the stoichiometry: Job’s method [38] and the complementary approach [37].

##### Job’s Method

The concentrations of both components (ferric ions and tested alkaloid) changed, while their concentration sum was kept constant. This was based on the principle that the highest quantity of the complex is formed under ideal conditions, which implies that under such conditions, the maximal absorbance is observed at the concentration ratio corresponding to the stoichiometry of the complex. The molar concentrations of alkaloid + ferric ions were kept constant at 100 μM, while their molar concentration ratios were continuously changed from 1:4 to 6:1. The measurements were performed against a blank composed of the buffer and methanol at a ratio of 2:1.

##### Complementary Method

For the complementary approach, the molar concentration of alkaloid was continuously changed, while the concentration of iron was kept constant throughout the series of samples (10 μM) with different molar concentration ratios, ranging from 1:4 to 6:1 (alkaloid:iron). The blank composition was the same as that used in the Job’s method. Based on the determined molar absorption coefficients and measured spectra, assessment of the stoichiometry was performed with construction of theoretical lines mimicking the absorbance of the most probable stoichiometries (see [37] for details).

#### 4.2.6. Statistical Analysis

The amount of non-chelated or reduced iron/copper ions was calculated from the difference in absorbance between the tested sample (with indicator) and its corresponding blank (without indicator) divided by the difference between the control sample (the known amount of iron/copper ions without the tested substance) and its control blank.

Normalized dose-dependent curves were constructed by use of GraphPad Prism version 7.03 for Windows by GraphPad Software (San Diego, CA, USA) for each condition from at least five points (first point 0% chelation, last point 100% chelation). The efficiency of chelation at concentration ratios of 10:1 and 1:1 (alkaloid:iron/copper) was calculated from the curve equation:

(1) $$y=\frac{100}{1+10^{\left(logEC50-x\right)*k}}$$

where *y* means the chelation efficiency in %, *x* is the common logarithm of concentration ratio of alkaloid:iron/copper and *k* is the slope of the curve.

Data are expressed as means ±SD. Results for individual alkaloids were compared with the standard chelators and the solvent by one-way ANOVA test followed by Bonferroni’s Multiple Comparison Test. Significant difference in reduction activity vs. the solvent (DMSO) was evaluated by *t*-test using MS Excel. Reduction lines were constructed by linear regression model from points, in which the reduction was significant vs. the solvent and the maximal reduction caused by the hydroxylamine. The 95% confidence intervals were created as well by GraphPad Software.

## 5. Conclusions

This study confirmed previous isolated results on the chelation effects of alkaloids. In particular, isoquinoline alkaloids with an *ortho* hydroxymethoxy site, a methylendioxy ring, or even an *ortho* dimethoxygroup are able to chelate ferrous or ferric ions under non-buffered conditions when DMSO is used as the solvent. However, most alkaloids lose their chelation effect in buffers and, in particular, with decreasing pH. At the same time, with the exception of protopines, most alkaloids are fairly potent reduction agents, particularly for cupric ions, at all the pathophysiologically relevant pH tested in this study (4.5–7.5). This paper opens new fields in this research as it is well known that metal chelation is or can be suitable in several pathological conditions, e.g., in cardiovascular disease and Alzheimer’s disease. On the other hand, metal reduction can potentiate the metal-based Fenton reaction, and this could render these compounds unsuitable in the treatment of these diseases but suitable for cancer treatment. Nevertheless, in compounds with chelation, reduction and, possibly, antioxidant effects, experimental data are crucial to define the final anti- or prooxidant effects.

## Data Availability

Upon request.

## Supplementary Materials

The following are available online at https://www.mdpi.com/article/10.3390/molecules27196429/s1, Figure S1. Structures of studied isoquinoline alkaloids. Figure S2. Stability of iron complexes. Figure S3. Illustrative examples of shift of glaucine spectra after addition of ferric ions. Figure S4. Examples of comparisons between reduction lines. Figure S5. Schematic simplified comparison of the most potent ferric reducing alkaloids at pH 4.5. Figure S6. Schematic simplified comparison of the most potent cupric-reducing alkaloids. Supplementary Table S1. Summarized data on the first absorption maxima of glaucine and its complexes with ferric ions. Supplementary Table S2. Significance of iron- and copper-reduction ability of alkaloids.

## Sample Availability

Samples of the compounds are available from the authors.
