# Supplementary material for: Interactions of Isoquinoline Alkaloids with Transition Metals Iron and Copper
Source: Molecules. 2022 Sep 29;27(19):6429. doi: 10.3390/molecules27196429 (PMC9572997; doi:10.3390/molecules27196429)
Supplement: Supplementary file 1 [file molecules-27-06429-s001.zip › molecules-1932208-supplementary.pdf]

Interactions of Isoquinoline Alkaloids with Transition Metals Iron and Copper

Mst Shamima Parvin, Jakub Chlebek, Anna Hošťálková,
Maria Carmen Catapano, Zuzana Lomozová, Kateřina Macáková
& Přemysl Mladěnka

SUPPLEMENTARY DATA

7 pages

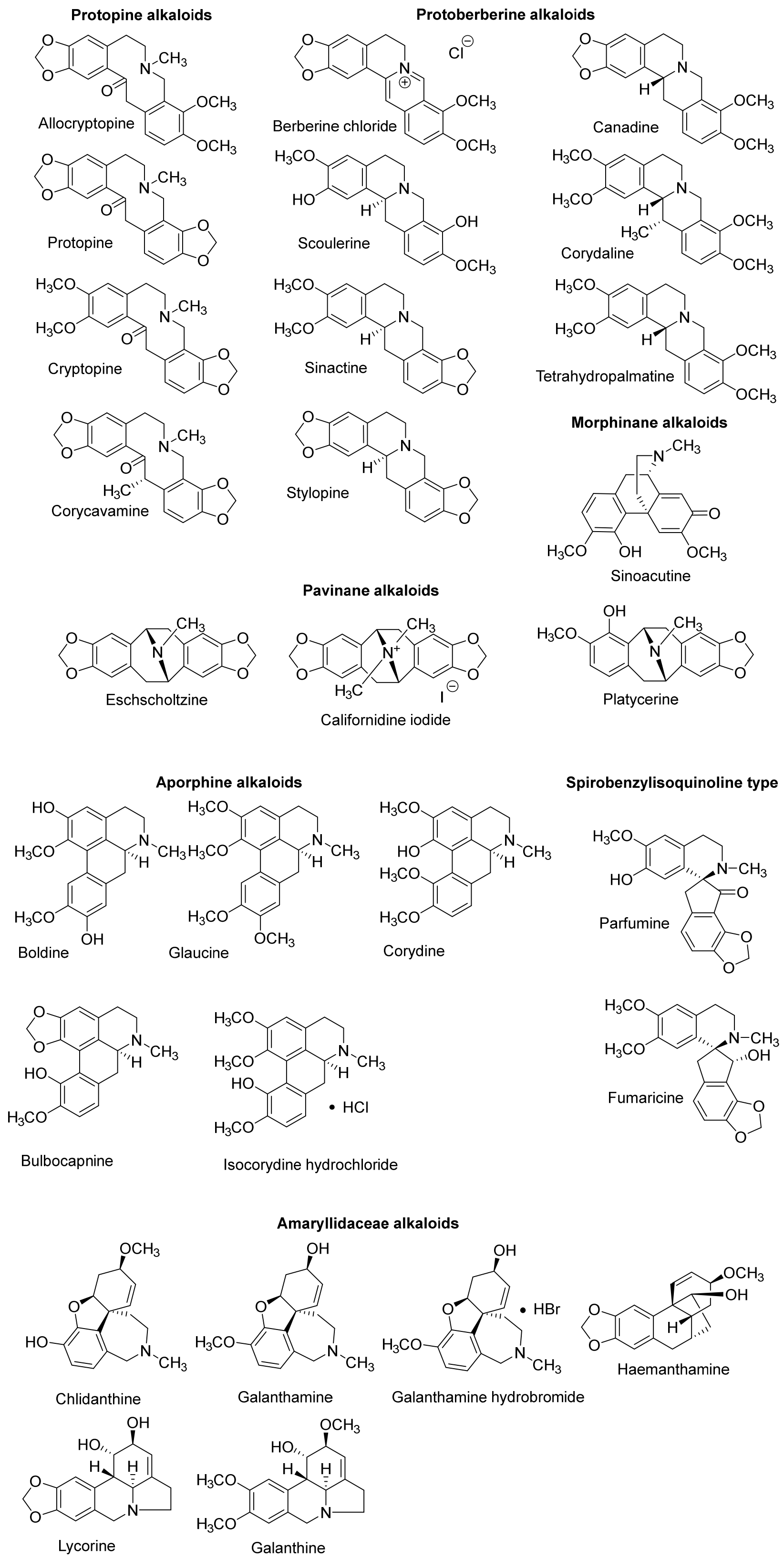

Supplementary Figure S1. Structures of studied isoquinoline alkaloids.

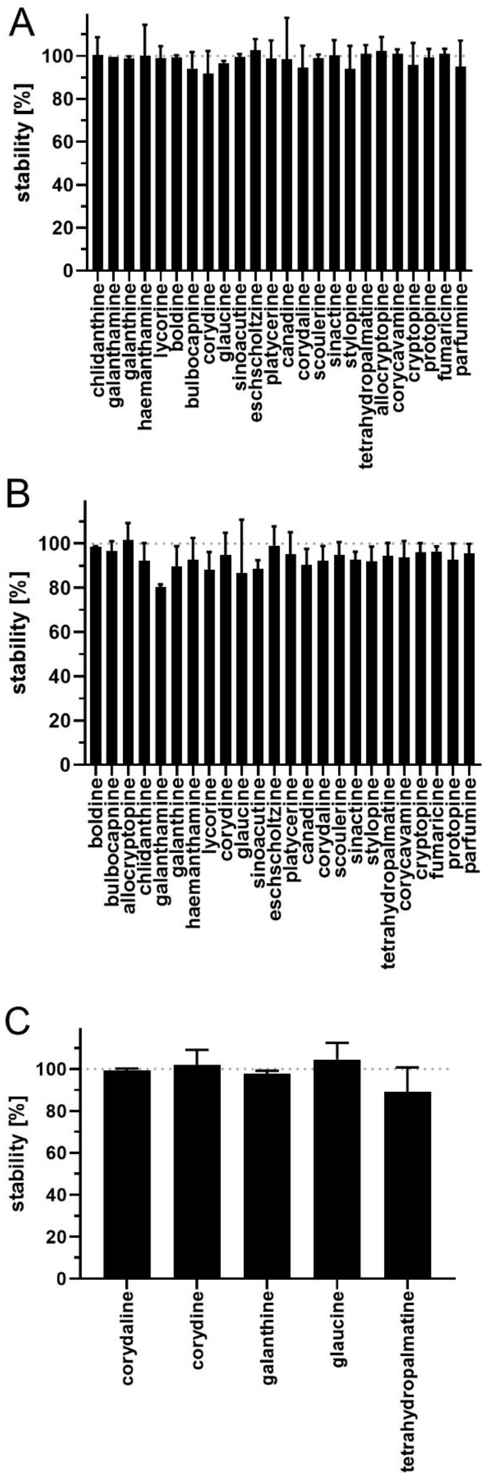

Supplementary Figure S2. Stability of iron complexes. A: ferrous complexes in non-buffered conditions (DMSO), **B**: ferric complexes under the same conditions, **C**: ferrous complexes at pH 7.5. Only alkaloid forming complexes under experimental conditions are shown. Stability was measured as a change of per cent chelation after 5 minutes *vs.* the measurement at time 0.

Supplementary Table S1. Summarized data on the first absorption maxima of glaucine and its complexes with ferric ions.

	**MeOH**	**pH 4.5**	**pH 5.5**	**pH 6.8**	**pH 7.5**
**λ_S [nm]**	221	224	221.5	228	225.5
**λ_C (Fe^{3+}) [nm]**	229.5	240	234.5	235	239.5

λs: absorbance maximum of the substance (glaucine).
λc: absorbance maximum of the complex.

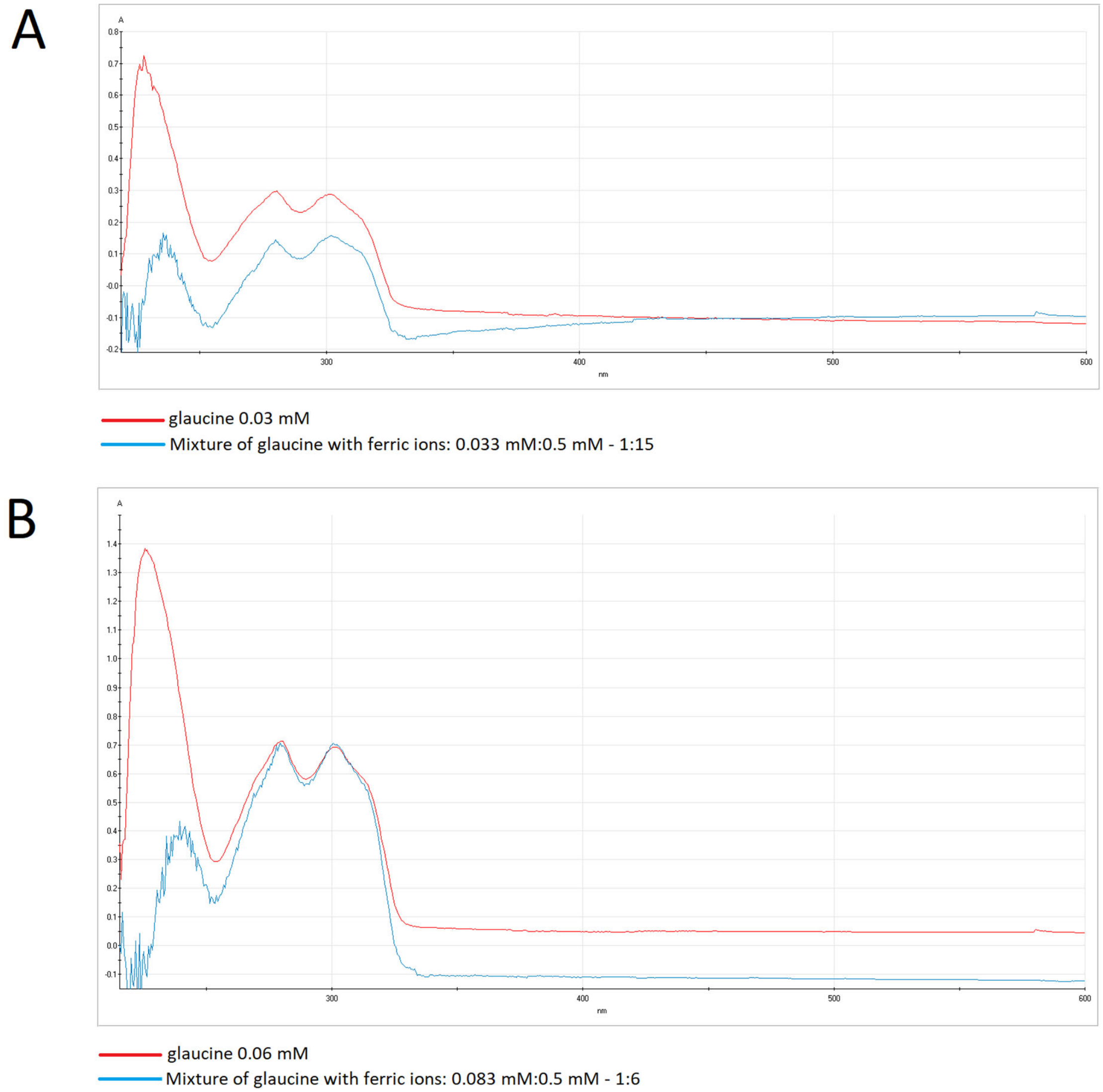

Supplementary Figure S3. Illustrative examples of shift of glaucine spectra after addition of ferric ions. Glaucine and Fe^{3+} pH 6.8 (**A**). Glaucine and Fe^{3+} pH 7.5 (**B**).

Supplementary Table S2. Significance of iron and copper reducing ability of alkaloids.

Substance	Iron reduction			Copper reduction				
	Non-buffered	pH 4.5	pH 5.5	Non-buffered	pH 4.5	pH 5.5	pH 6.8	pH 7.5
Amaryllidaceae alkaloids								
chlidanthine	n.s.	<0.001	n.s.	<0.001	<0.001	<0.01	<0.001	<0.001
galanthamine hydrobromide	<0.001	<0.01	n.s.	<0.001	<0.001	<0.01	<0.01	<0.01
galanthamine	n.s.	n.s.	n.s.	<0.001	<0.01	<0.01	<0.001	<0.001
galanthine	n.s.	<0.01	n.s.	<0.001	<0.01	<0.01	<0.01	<0.001
haemanthamine	n.s.	n.s.	n.s.	<0.01	<0.001	<0.001	<0.01	<0.05
lycorine	n.s.	<0.001	n.s.	<0.001	<0.01	<0.01	<0.01	<0.001
aporphine alkaloids								
boldine	<0.01 aprox. 2:1	<0.001	n.s.	<0.001	<0.001	<0.001	<0.001	<0.001
bulbocapnine	<0.01 aprox. 1:1	<0.01	n.s.	<0.001	<0.001	<0.001	<0.001	<0.001
corydine	<0.01 aprox. 1:1	<0.01	n.s.	<0.001	<0.01	<0.01	<0.05	<0.01
glaucine	<0.001	<0.001	<0.01	<0.001	<0.001	<0.001	<0.01	<0.01
isocorydine chloride	<0.01 aprox. 1:1	<0.001	n.s.	<0.001	<0.001	<0.001	<0.01	<0.001
morphinane alkaloid								
sinoacutine	<0.001 aprox. 1:1	<0.001	n.s.	<0.001	<0.001	<0.001	<0.01	<0.01
pavinane alkaloids								
californidine iodide	<0.001	n.s.	n.s.	<0.001	<0.001	<0.01	<0.05	<0.05
eschscholtzine	n.s.	<0.001	n.s.	<0.001	<0.01	<0.001	<0.05	<0.01
platycerine	n.s.	<0.01	n.s.	<0.001	<0.001	<0.01	<0.05	<0.01
protoberberine alkaloids								
berberin chloride	<0.001	<0.001	n.s.	<0.001	<0.001	<0.001	<0.01	<0.01
canadine	n.s.	<0.05	n.s.	<0.05	<0.01	n.s.	n.s.	n.s.
corydaline	n.s.	n.s.	n.s.	<0.01	<0.05	<0.05	<0.05	<0.001
scoulerine	<0.001 aprox. 1:1	<0.001	n.s.	<0.001	<0.001	<0.001	<0.01	<0.001
sinactine	n.s.	<0.001	n.s.	<0.01	<0.01	<0.001	<0.001	<0.01
stylopine	n.s.	n.s.	n.s.	<0.001	<0.05	n.s.	n.s.	n.s.
tetrahydropalmatine	n.s.	<0.05	n.s.	<0.001	<0.01	<0.01	<0.05	<0.05
protopine alkaloids								
allocryptopine	n.s.	n.s.	n.s.	n.s.	n.s.	n.s.	n.s.	n.s.
corycavamine	n.s.	n.s.	n.s.	n.s.	n.s.	n.s.	n.s.	n.s.
cryptopine	n.s.	n.s.	n.s.	n.s.	n.s.	n.s.	n.s.	n.s.
protopine	n.s.	n.s.	n.s.	n.s.	n.s.	n.s.	n.s.	n.s.
spirobenzylisoquinoline alkaloids								
fumaricine	n.s.	n.s.	n.s.	<0.001	n.s.	<0.001	<0.05	<0.05
parfumine	n.s.	<0.001	n.s.	<0.01	<0.001	<0.001	<0.01	<0.001

Significant differences in reduction *vs.* the solvent at the highest concentration ratio (alkaloid:iron), unless otherwise stated. The table shows the p values calculated by the t-test. n.s.; non-significant.

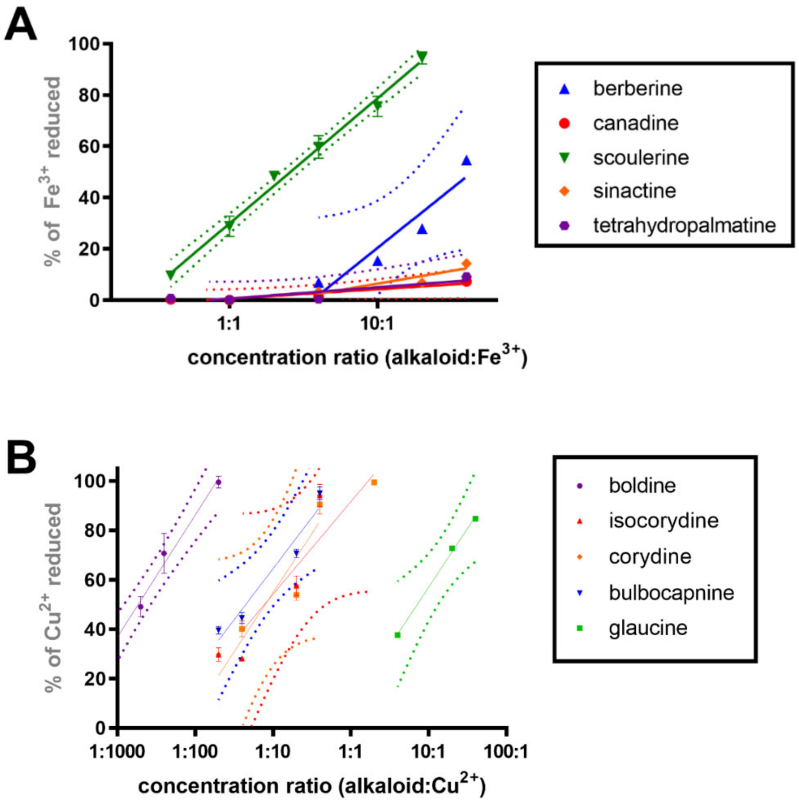

Supplementary Figure S4. Examples of comparisons between reduction lines. **A**: The figure shows ferric reduction of protoberberines by reduction lines and 95% confidence intervals at pH 4.5. **B**: The figure shows cupric reduction of aporphines by reduction lines and 95% confidence intervals at pH 7.5.

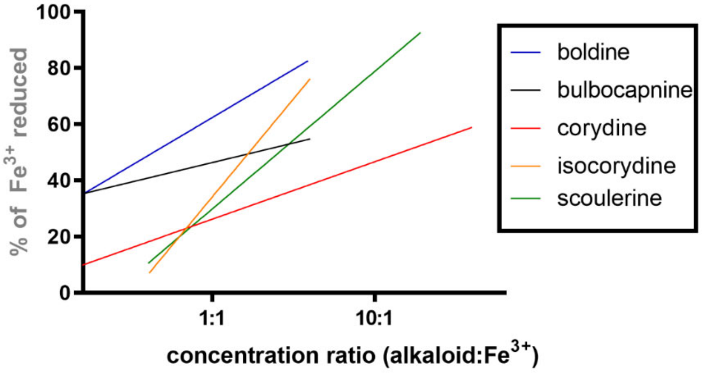

Supplementary Figure S5. Schematic simplified comparison of the most potent ferric ions reducing alkaloids at pH 4.5. All these alkaloids were able to reduce the iron in ratios lower 1:1. For reason of lucidity, only reduction lines are shown.

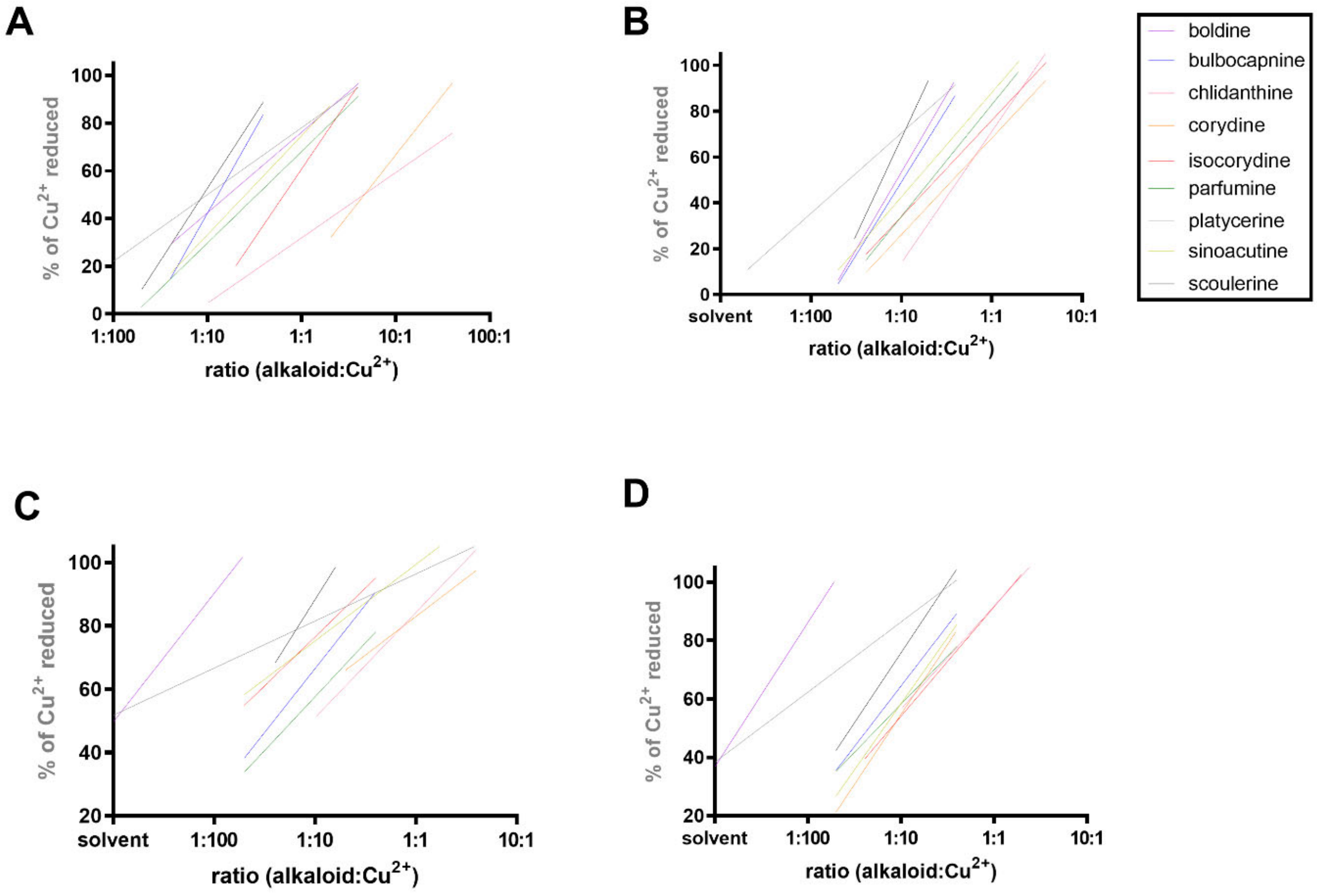

Supplementary Figure S6. Schematic simplified comparison of the most potent cupric ions reducing alkaloids. For reason of lucidity, only reduction lines are shown. **A**: pH 4.5, **B**: pH 5.5, **C**: pH 8.8 and **D**: pH 7.5.
